# Acoustic Bessel Vortex Beam by Quasi-Three-Dimensional Reflected Metasurfaces

**DOI:** 10.3390/mi12111388

**Published:** 2021-11-12

**Authors:** Yin Wang, Jiao Qian, Jian-Ping Xia, Yong Ge, Shou-Qi Yuan, Hong-Xiang Sun, Xiao-Jun Liu

**Affiliations:** 1 Research Center of Fluid Machinery Engineering and Technology, School of Physics and Electronic Engineering, School of Computer Science and Communication Engineering, Jiangsu University, Zhenjiang 212013, China; 2112008007@stmail.ujs.edu.cn (Y.W.); jiao.qian@duke.edu (J.Q.); jianping.xia@duke.edu (J.-P.X.); geyong@ujs.edu.cn (Y.G.); shouqiy@ujs.edu.cn (S.-Q.Y.); 2 Collaborative Innovation Center of Advanced Microstructures, Key Laboratory of Modern Acoustics, Department of Physics, Nanjing University, Nanjing 210093, China; 3 State Key Laboratory of Acoustics, Institute of Acoustics, Chinese Academy of Sciences, Beijing 100190, China

**Keywords:** acoustic wave, vortex beam, Bessel beam, acoustic metasurfaces, phase modulation

## Abstract

Vortex beams have a typical characteristic of orbital angular momentum, which provides a new degree of freedom for information processing in remote communication and a form of non-contact manipulation for trapping particles. In acoustics, vortex beams are generally observed on the surface of a metamaterial structure or in a waveguide with a hard boundary owing to the characteristic of easy diffusion in free space. The realization of an acoustic vortex beam with a long-distance propagation in free space still remains a challenge. To overcome this, we report a type of acoustic Bessel vortex (ABV) beam created by a quasi-three-dimensional reflected metasurface in free space based on phase modulation. By using the Bessel and vortex phase profiles, we can realize an ABV beam with the high performances of both Bessel and vortex beams, and its effective propagation distance is larger than 9.2*λ* in free space. Beyond that, we discuss the bandwidth and topological charge of the ABV beam in detail, and the fractional bandwidth can reach about 0.28. The proposed ABV beam has the advantages of a high-performance vortex, long-distance propagation, and broad bandwidth, which provide a new pathway for designing multifunctional vortex devices with promising applications.

## 1. Introduction

Recent years have witnessed the great development of acoustic vortex beams owing to their extensive applications in a wide range of fields, such as sound communication [1,2] and particle trapping [3,4,5]. The acoustic vortex beam can transfer different-order vortex wavefronts with orbital angular momentum, opening up a new degree of freedom for sound modulations [6,7,8,9,10]. Traditionally, by designing active sound arrays composed of phased sources [2,11,12,13], researchers have experimentally realized acoustic vortex beams to manipulate micro-particles underwater. However, the high cost and complex circuits of the active systems create some difficulties for their further applications.

To overcome these restrictions, the emergence of acoustic metamaterials [14,15,16,17,18,19,20] and metasurfaces [21,22,23,24,25,26,27] have provided alternative design schemes for passive acoustic lenses of vortex beams. As examples, by designing thickness-gradient structures [28,29] or an acoustic plate with a spiral shaped structure [30,31,32], the acoustic vortex beam can be observed around structure surfaces. However, their structures must satisfy the geometric characteristics of spiral distribution. On the other hand, by tailoring phase profiles of acoustic metasurfaces, sound energy can also be formed as an acoustic vortex beam based on phase modulation [33,34,35,36,37]. For instance, by designing phase profiles of Helmholtz resonators in a circular waveguide, acoustic vortex beams are also observed, and the sound energy of vortex beams can propagate in the waveguide with a hard boundary [34,37]. Furthermore, multifunctional vortex beams of sound can also be obtained by changing phase profiles, such as focusing vortices [38,39] and vortex beams with asymmetric propagation [40], which have potential special applications. Additionally, the finite element method based on the COMSOL Multiphysics software has been introduced to numerically design and optimize different types of acoustic vortex devices, such as unidirectional vortex beams through acoustic Weyl crystal with a topological lattice defect, and a vortex converter composed of an acoustic metagrating in a waveguide [10,37,40], and the corresponding simulated results agree well with the measured ones. However, these aforementioned vortex beams are generally observed on structure surfaces or in waveguides with a hard boundary owing to the characteristic of easy diffusion in free space. The realization of an acoustic vortex beam with long-distance propagation in free space still poses a great challenge.

In our work, we propose a type of quasi-three-dimensional (quasi-3D) reflected metasurface to realize an acoustic Bessel vortex (ABV) beam by phase modulation. Based on the Bessel and vortex phase profiles, we can realize an ABV beam with the advantages of both a high-performance vortex and long-distance propagation in free space, which overcomes the limitations of easy diffusion in free space. Moreover, we discuss the working bandwidth of the ABV beam in detail, and its fractional bandwidth can reach about 0.28, showing the characteristic of broad bandwidth. Finally, we design and realize ABV beams with different topological charges. The designed ABV beams with a high-performance vortex and long-distance propagation may lead to important advances in diverse applications.

## 2. Design and Methods

**Figure 1:**
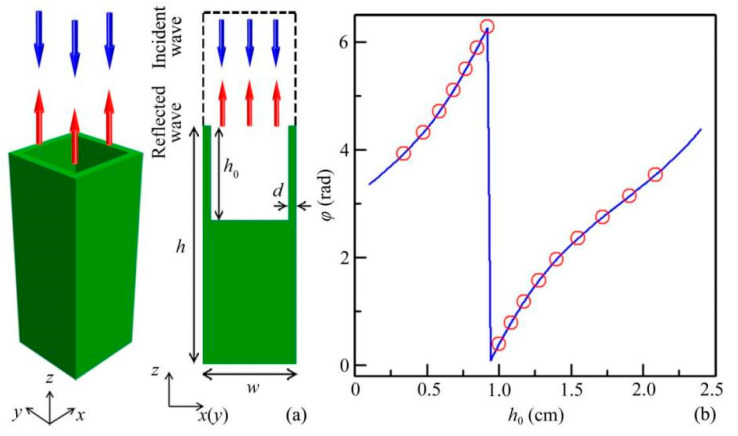
Design of a quasi-3D reflected unit. (**a**) Schematic of a quasi-3D reflected unit and its *x*(*y*)-*z* cross section. (**b**) Reflected phase delays created by the quasi-3D reflected unit as a function of tunable depth *h*_0_.

As schematically shown in Figure 1a, we propose a type of quasi-3D reflected unit composed of a cuboid solid with a groove on the upper side. The cross sections *x*-*z* and *y*-*z* of the unit are the same, and the structure parameters are selected as *w* = 1.0 cm, *h* = 2.5 cm, and *d* = 0.1 cm. The groove depth *h*_0_ is tunable to modulate reflected phase delays of sound. The incident wavelength *λ* of sound is selected as 3.8 cm (viz., frequency *f*_0_ = 9.0 kHz in air). Throughout this work, the characteristics of sound reflections are numerically simulated by the COMSOL Multiphysics software. Here, the pressure field is calculated in the acoustic–solid interaction module, and the solid structure is made of epoxy resin to satisfy the acoustic-structure boundary conditions. The material parameters are adopted as follows: the density *ρ* = 1180 kg/m^3^, the longitudinal wave velocity *c*_l_ = 2720 m/s, and the transversal wave velocity *c*_t_ = 1460 m/s for epoxy resin; *ρ* = 1.21 kg/m^3^ and the sound velocity *c* = 343 m/s for air.

Figure 1b shows the reflected phase delays of sound created by the quasi-3D units with different values of *h*_0_. We can see that, in the range 0.1 < *h*_0_ < 2.4 cm, the theoretical reflected phase delay (blue solid line) can cover the whole 2π range. Here, we select 16 discrete values of *h*_0_ (red open circles) to realize an equally spaced phase delay from 0 to 2π with a step of π/8.

**Figure 2:**
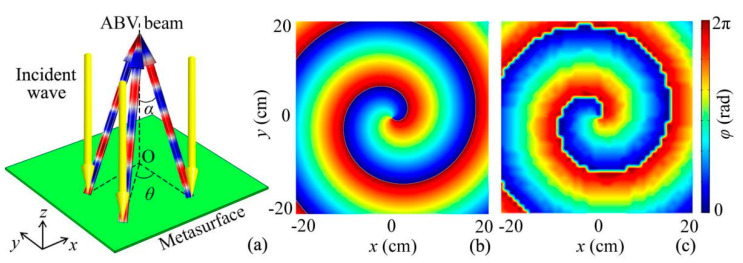
Design of the reflected ABV beam. (**a**) Schematic of a quasi-3D reflected metasurface of ABV beam, and its (**b**) theoretical continuous and (**c**) discrete phase profiles.

Based on the selected 16 reflected phased units, we can design a type of planar quasi-3D reflected metasurface of the ABV beam by phase modulation. Figure 2a shows the normal incidence of an acoustic wave on the quasi-3D reflected metasurface, and the sound reflections are formed as an ABV beam. To design it, we introduce the phase profile of a vortex beam in free space, which is expressed as

(1) $$\varphi_{s}(\theta )=n\theta ,$$

where *n* is the topological charge of the vortex beam [31], and *θ* is the azimuth angle which is in the range from 0 to 2π in the *x*-*y* plane. To realize a vortex beam with long-distance propagation in free space, we here introduce a non-diffracting Bessel phase profile. Based on the 3D generalized Snell’s law [41] and Equation (1), the final phase profile of the designed quasi-3D reflected metasurface in the *x-y* plane is given as

(2) $$\varphi =-k\sin\alpha \sqrt{x^{2}+y^{2}}+n\theta ,$$

where *k* = 2π*f*/*c* is the wave number in air, and *c* and *f* are the sound velocity and the frequency, respectively. *α* is the base angle of the Bessel beam, and the parameter *θ* satisfies tan *θ* = *x*/*y*. Here, the parameters *α* and *n* are selected as 15° and 1, respectively, and the size of the quasi-3D metasurface in the *x*-*y* plane is 40 cm × 40 cm. Figure 2b,c show the theoretical continuous and discrete phase delays of the quasi-3D reflected metasurface in the *x*-*y* plane, respectively. It is worth noting that the phase distribution shows characteristics typical of a vortex beam, and both types of phase profiles agree well with each other.

## 3. Results and Discussion

### 3.1. Performance of ABV Beam

**Figure 3:**
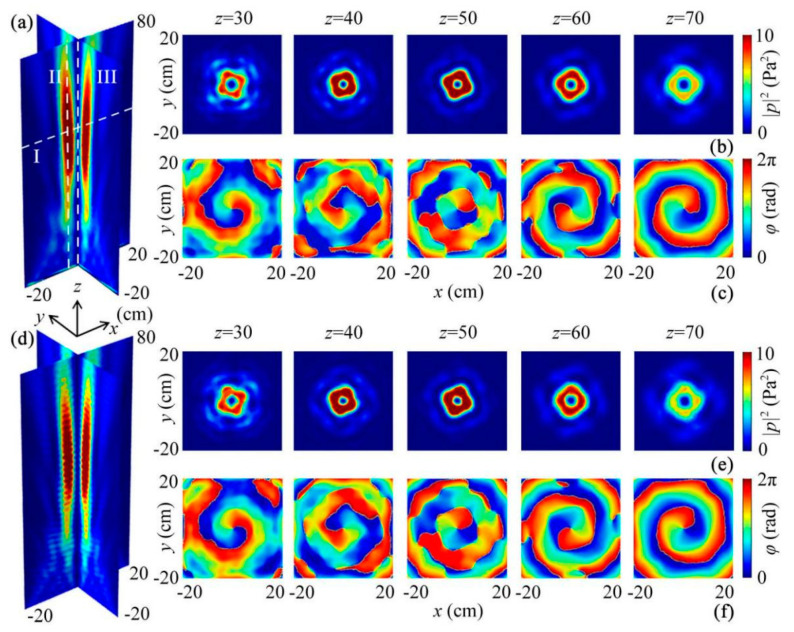
The performance of the reflected ABV beam with *n* = 1 at 9.0 kHz. (**a**) Simulated 3D reflected intensity distribution of the ABV beam created by the quasi-3D metasurface, and its corresponding reflected (**b**) intensity and (**c**) phase distributions at 5 selected cross sections *z* = 30, 40, 50, 60, and 70 cm. (**d**) Simulated 3D reflected intensity distribution of the ABV beam created by the theoretical continuous phase profile, and its corresponding reflected (**e**) intensity and (**f**) phase distributions at the 5 cross sections.

Based on the two types of phase profiles, we realized an ABV beam by designing a quasi-3D reflected metasurface. Figure 3a shows the simulated 3D reflected intensity distribution created by the designed quasi-3D metasurface composed of 16 types of phased units at 9.0 kHz. We observed that the sound reflections were concentrated into a long-distance hollow cylinder along the *z* direction, which can cover a range from 28 to 72 cm (|*p*|^2^ > 5.0 Pa^2^). On the other hand, the energy of the sound beam in the central region was almost zero, showing typical features of both Bessel and vortex beams. To further show the characteristics of the ABV beam, we also displayed the reflected acoustic intensity and phase distributions of the ABV beam at 5 selected cross sections (*z* = 30, 40, 50, 60, and 70 cm), which are shown in Figure 3b,c, respectively. It is obvious that, with the increase of *z*, there existed a concentric ring-shaped region at the center of each cross section (Figure 3b), which was the same as that in Figure 3a. Beyond that, the sound energy in the ring-shaped region (|*p*|^2^ > 6.0 Pa^2^) was much larger than the central energy of sound (|*p*|^2^ ≈ 0) at the 5 cross sections owing to the existence of a phase singularity at the center (Figure 3c). The phase distributions in Figure 3c show the typical characteristics of the vortex beam with *n* = 1, further verifying that the ABV beam had both advantages of long-distance propagation and a high-performance vortex in free space. In addition to the aforementioned results, we simulated the acoustic intensity and phase distributions created by the theoretical continuous phase profile for comparison, which are shown in Figure 3d–f. We can see that the characteristics of the observed ABV beam matched well with those in Figure 3a–c, which further demonstrates the performance of the reflected ABV beam by the quasi-3D metasurface.

**Figure 4:**
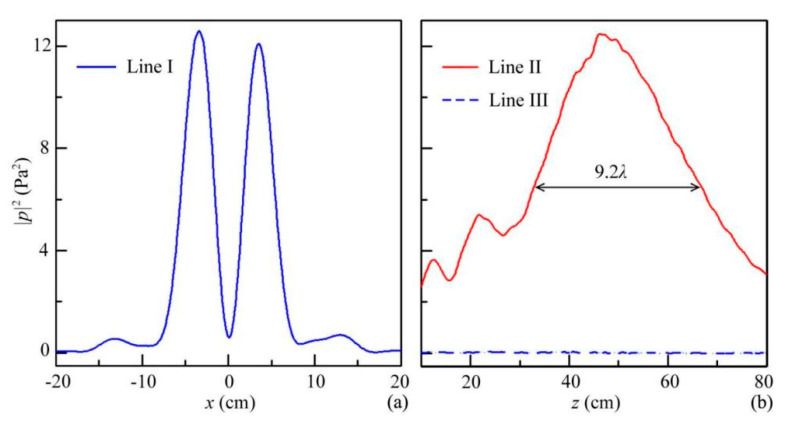
Simulated acoustic intensity distributions through the ABV beam along lines (**a**) I, (**b**) II, and III in Figure 3d.

To quantify the performances of the ABV beam, we also display the simulated longitudinal and transverse intensity distributions of the ABV beam (the lines I–III in Figure 3a), which are shown in Figure 4. We observe that two acoustic energy peaks (|*p|*^2^ ≈ 12.0 Pa^2^) existed on both sides of *x* = 0, while the sound intensity at *x* = 0 was close to zero (Figure 4a). In addition, as shown in Figure 4b, the sound intensities along line II were much larger than those along line III (|*p*|^2^ ≈ 0), which arose from the existence of the phase singularity along line III. Moreover, the total width at half maximum of the sound intensity along line II reached about 35 cm (9.2*λ*), showing that the energy of the ABV beam can propagate a long distance in free space. Therefore, we further verified the high performances of both Bessel and vortex beams for the ABV beam created by the quasi-3D reflected metasurface.

### 3.2. Bandwidth of ABV Beam

**Figure 5:**
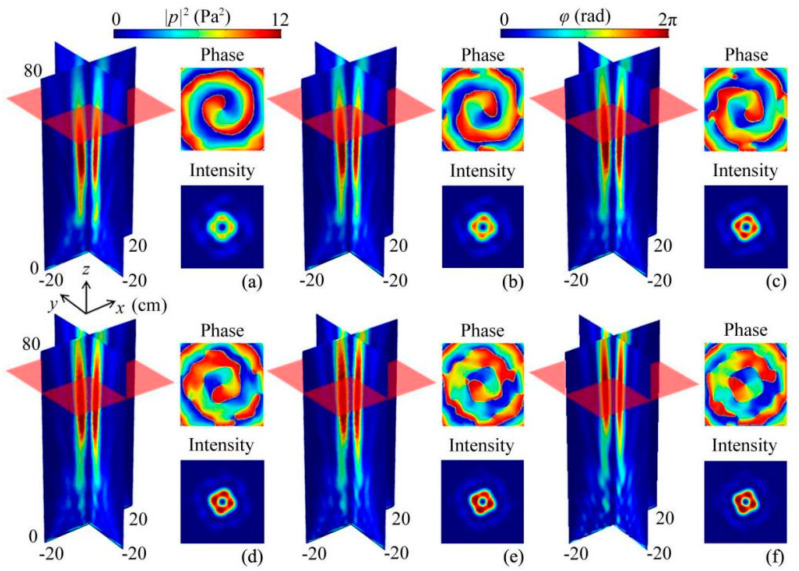
Simulated 3D reflected intensity distributions of the ABV beam created by the quasi-3D metasurface at (**a**) 8.2, (**b**) 8.7, (**c**) 9.2, (**d**) 9.7, (**e**) 10.2, and (**f**) 10.7 kHz. Insets on the right side represent the phase and intensity distributions at a cross section *z* = 60 cm (red translucent planes).

Next, we discuss the bandwidth of the quasi-3D reflected metasurface of the ABV beam. Figure 5 shows the intensity and phase distributions created by the quasi-3D reflected metasurface at different frequencies, in which the other parameters remain unchanged. It is noted that, with the increase of frequency, the center position of the ABV beam moved along the +*z* direction gradually. However, the characteristics of the ABV beam stayed the same as those in Figure 3d–f. Moreover, we also simulated the intensity and phase distributions of the ABV beam at both edge frequencies (8.0 and 11.0 kHz), which are shown in Section Note 1 of the Supplementary Materials. Therefore, the working bandwidth of the designed ABV beam can exceed 2.5 kHz, and its fractional bandwidth is larger than 0.28, showing a broadband characteristic.

### 3.3. Design of ABV Beam with Different Values of n

**Figure 6:**
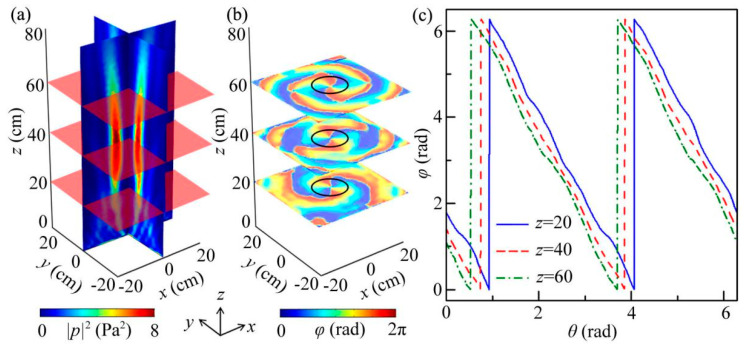
The performance of the reflected ABV beam with *n* = 2 at 9.0 kHz. (**a**) Simulated 3D reflected intensity distribution of the ABV beam created by the quasi-3D metasurface, and its corresponding reflected (**b**) phase distribution at 3 cross sections *z* = 20, 40, and 60 cm. (**c**) Phase delays around in a circle with the radius of 6 cm (black open circles in (**b**)) as a function of *θ* at the 3 cross sections.

Based on Equation (2), we designed the quasi-3D reflected metasurfaces of the ABV beam with different values of *n*, and their theoretical continuous and discrete phase delays in the *x*-*y* plane are shown in Section Note 2 of the Supplementary Materials. Here, we selected *n* = 2 as an example, and the simulated intensity and phase distributions created by the quasi-3D reflected metasurface at 9.0 kHz, which are shown in Figure 6a,b, respectively. We observe that the sound reflections were concentrated into a hollow cylinder region with a height of 9*λ*, and the characteristics of the ABS beam were similar to those in Figure 3d (*n* = 1). However, for the phase distribution in Figure 6b, we observe that the phase delay shifted from 0 to 2π twice around in a circle at 3 cross sections *z* = 20, 40 and 60 cm, which was different from that in Figure 3f (*n* = 1). Such a phenomenon is also obviously displayed in Figure 6c. Beyond that, we also designed a fractional ABV beam with values of *n* = 1.25, 1.5, and 1.75, which is shown in Section Note 3 of the Supplementary Materials. Therefore, by combining both phase profiles of Bessel and vertex beams, we realized an ABV beam with different topological charges.

### 3.4. Design of Underwater ABV Beam

Finally, we designed a type of planar quasi-3D reflected metasurface of an underwater ABV beam, and simulated the intensity distribution of the underwater ABV beam at 30 kHz, which is shown in Section Note 4 of the Supplementary Materials. The results show that the simulated underwater ABV beam also exhibited characteristics typical of a vortex beam, which can cover a range from 30 to 120 cm (|*p*|^2^ > 2.5 Pa^2^) along the *z* direction. The propagation distance of the underwater ABV beam (about 22*λ*) was larger than that (about 10*λ*) of the holographic algorithm [42]. The designed underwater ABV beam with long-distance propagation will be important for realizing advanced in vivo acoustic tweezers, as it can penetrate deeper into a lesion location, which can be potentially applied to removing broken bones, thrombus ablation, and nerve stimulation.

## 4. Conclusions

In conclusion, we have demonstrated a quasi-3D reflected metasurface of an ABV beam. The results show that, by using the Bessel and vortex phase profiles, we realized an ABV beam with a high-performance vortex, and its effective propagation distance can exceed 9.2*λ* in free space, which overcomes the restriction of easy diffusion of vortex beams propagating in free space. Additionally, the designed ABV beam had the characteristic of broad bandwidth, and its fractional bandwidth reached about 0.28. Finally, by modulating the phase profile of the quasi-3D reflected metasurface, we observed ABV beams with different topological charges, where *n* = 1.25, 1.5, 1.75, and 2. In contrast to the characteristics of acoustic vortex beams [31,34], the proposed ABV beam with the features of long-distance propagation, a high-performance vortex and broad bandwidth provides a novel methodology for sound manipulations and a fertile ground for designing multifunctional vortex devices, which also play an important role in the field of underwater structure measurement.

## Supplementary Materials

The following are available online at https://www.mdpi.com/article/10.3390/mi12111388/s1, Note 1: Intensity and phase distributions of ABV beam at both edge frequencies. Note 2: Theoretical continuous and discrete phase delays of the quasi-3D reflected metasurface with different values of *n*. Note 3: Intensity and phase distributions of fractional reflected ABV beam. Note 4: Design of underwater ABV Beam
