# Supplementary material for: Acoustic Bessel Vortex Beam by Quasi-Three-Dimensional Reflected Metasurfaces
Source: Micromachines (Basel). 2021 Nov 12;12(11):1388. doi: 10.3390/mi12111388 (PMC8623545; doi:10.3390/mi12111388)
Supplement: Supplementary file 1 [file micromachines-12-01388-s001.zip › micromachines-1453902-supplementary.pdf]

Supplementary Material For

Acoustic Bessel vortex beam by quasi-three-dimensional reflected metasurfaces

Yin Wang[1†], Jiao Qian[1†], Jian-Ping Xia[1†], Yong Ge[1], Shou-Qi Yuan[1], Hong-Xiang Sun[1,2,3*] and Xiao-Jun Liu[2, 3*]

[1] Research Center of Fluid Machinery Engineering and Technology, School of Physics and Electrical Engineering, School of Computer Science and Communication Engineering, Jiangsu University, Zhenjiang 212013, China

[2] Key Laboratory of Modern Acoustics, Department of Physics and Collaborative Innovation Center of Advanced Microstructures, Nanjing University, Nanjing 210093, China

[3] State Key Laboratory of Acoustics, Institute of Acoustics, Chinese Academy of Sciences, Beijing 100190, China

[*] Correspondence: jsdxshx@ujs.edu.cn; liuxiaojun@nju.edu.cn

[†] These authors contributed equally to this work

Note 1 Intensity and phase distributions of ABV beam at both edge frequencies.........2

Note 2 Theoretical continuous and discrete phase delays of the quasi-3D reflected metasurface with different values of *n* 2

Note 3 Intensity and phase distributions of fractional reflected ABV beam.................3

Note 4 Design of underwater ABV Beam..4

Note 1 Intensity and phase distributions of ABV beam at both edge frequencies

Figure S1a,b show the simulated intensity and phase distributions of the ABV beam at 8.0 and 11.0 kHz, respectively. It is obvious that the observed ABV beam has basic characteristics of both Bessel and vortex beams at both edge frequencies, indicating the working bandwidth of the ABV beam can reach about 3.0 kHz.

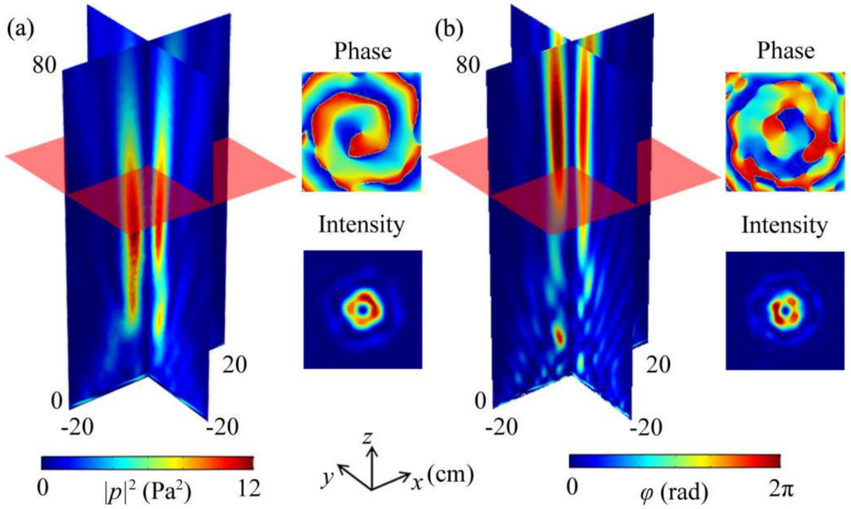

Figure S1. Simulated 3D reflected intensity distributions of the ABV beam created by the quasi-3D reflected metasurface at (**a**) 8.0 and (**b**) 11.0 kHz. Insets on the right side represent the phase and intensity distributions at a cross section z=55 cm (red translucent planes).

Note 2 Theoretical continuous and discrete phase delays of the quasi-3D reflected metasurface with different values of *n*

Figure S2a,b show the theoretical continuous and discrete phase delays of the quasi-3D reflected metasurfaces with n=1.25, 1.5, 1.75, and 2, respectively. we can see that the phase distributions show typical characteristics of the vortex beam, and both types of phase profiles agree well with each other.

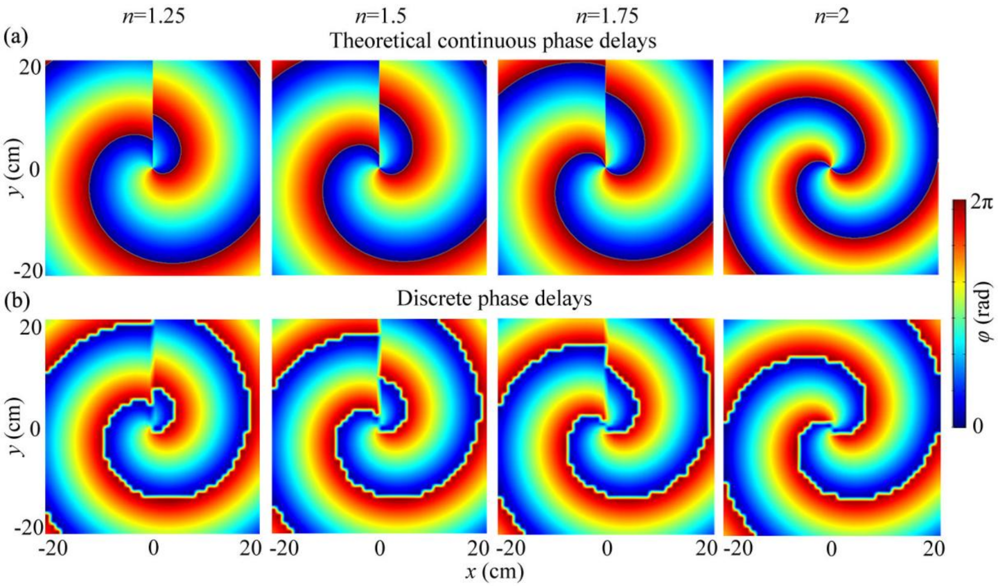

Figure S2. (a)Theoretical continuous and (b) discrete phase delays of the quasi-3D reflected metasurfaces with n=1.25, 1.5, 1.75, and 2, respectively.

Note 3 Intensity and phase distributions of fractional reflected ABV beam

Figure S3a-c show the simulated reflected 3D acoustic intensity and phase distributions of the ABV beam with different topological charge n=1.25, 1.5 and 1.75, respectively. We find that the characteristics of the fractional ABV beams are similar to those in Figure 3a. However, it is noted that the fractional ABV beams have nonuniform intensity distributions owing to its fractional topological charges. Besides, the phase distributions of these fractional ABV beams at a cross section z=60 cm also exhibit the characteristic of high-performance vortex [insets on the bottom side in Figure S3a-c], and their phase delays change 1.25π (blue solid line), 1.5π (red dotted line), and 1.75π (green dotted dashed line) around a circle with a radius of 6 cm for n= 1.25, 1.5, and 1.75, respectively [Figure S3d], showing obvious characteristics of fractional vortex beams.

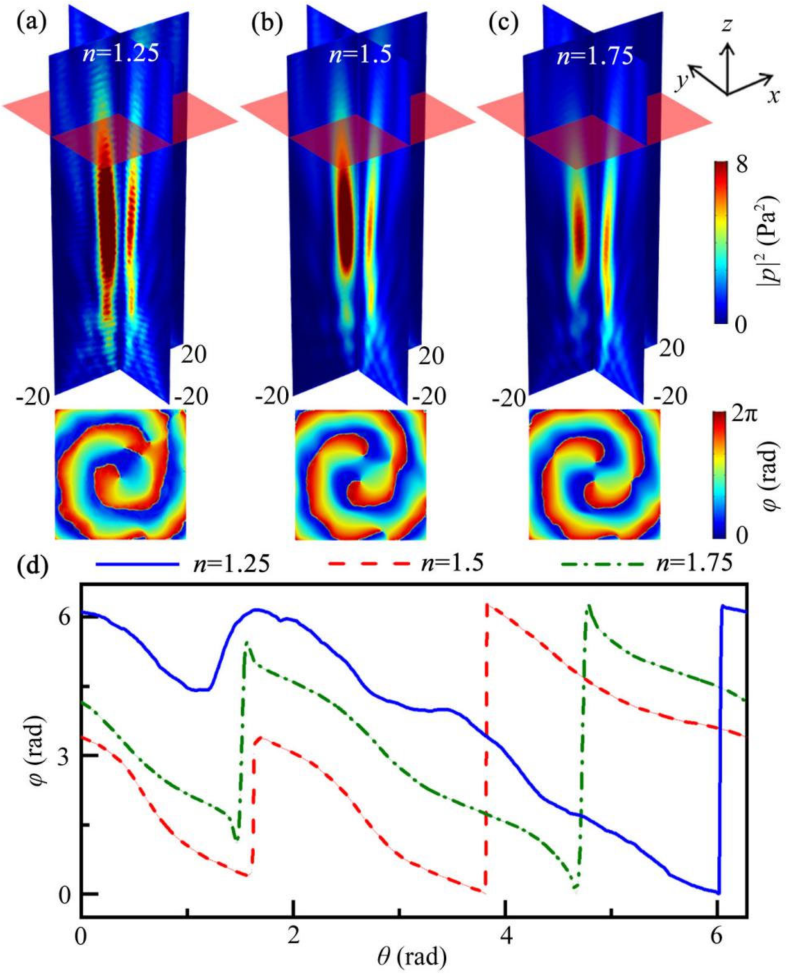

Figure S3. Intensity and phase distributions of fractional reflected ABV beam. Reflected 3D acoustic intensity and phase distributions and of the fractional ABV beams with the topological charge n= (**a**) 1.25, (**b**) 1.5, and (**c**) 1.75. Insets on the bottom side represent the phase distributions at a cross section z=60 cm (red translucent planes), and (**d**) their phase delays around in a circle (the radius of 6cm) as a function of θ.

Note 4 Design of underwater ABV Beam

To realize underwater ABV Beam, we introduce a type of quasi-3D reflected unit made of brass to design a quasi-3D reflected metasurface in water. The cross section of the unit is shown in Figure S4a, and its structure parameters are the same as those in Figure 1a. The material parameters used in the simulation are the density ρ=1000 kg/m^3 and the sound velocity c=1490 m/s for water; and the density ρ=8800 kg/m^3, the Young modulus E=108.42 GPa, and the Poisson ratio v=0.33 for brass. Figure S4b

shows the reflected phase delays of sound created by the quasi-3D units with different values of h_0, and its theoretical reflected phase delay (blue solid line) can cover the whole 2π range in the range $1.0<h_0<2.0$ cm. Here, we also select 16 discrete values of h_0 (red open circles) to realize an equally spaced phase delay from 0 to 2π with a step of $\pi/8$.

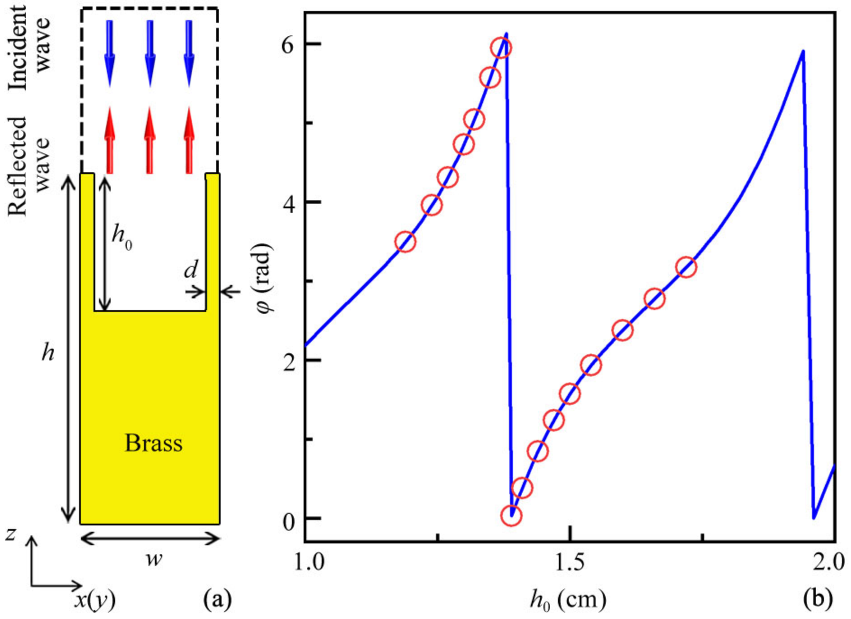

Figure S4. (**a**) Schematic of $x(y)$-z cross section of a quasi-3D reflected unit made of brass immersed in water, and its (**b**) reflected phase delays as a function of tunable depth h_0 at 30 kHz.

Based on the same design method, we design a type of planar quasi-3D reflected metasurface of the underwater ABV beam, which is shown in the Figure S5a. Here, the size of the quasi-3D metasurface is same as that in Figure 2a, and its theoretical continuous and discrete phase delays of in the x-y plane are shown in Figure S5b,c, respectively.

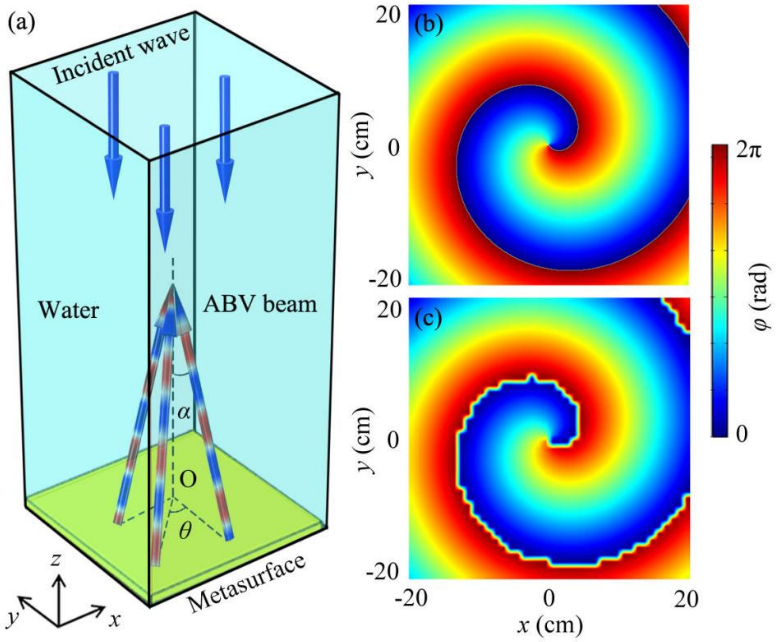

Figure S5. (**a**) Schematic of a quasi-3D reflected metasurface of underwater ABV beam, and its (**b**) theoretical continuous and (**c**) discrete phase profiles at 30 kHz.

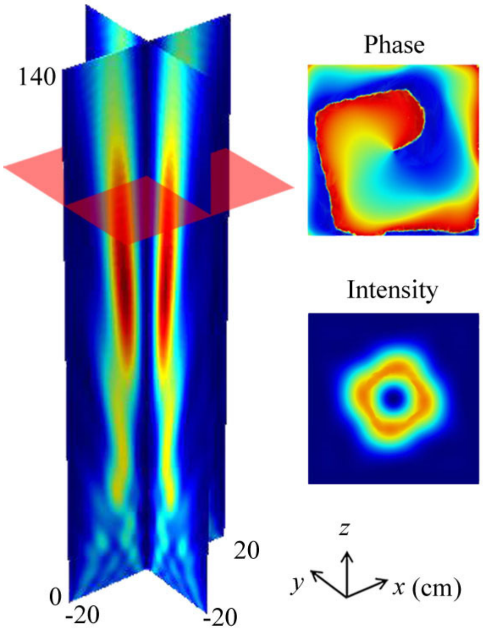

Figure S6. Simulated 3D reflected intensity distributions of the underwater ABV beam created by the quasi-3D reflected metasurface at 30 kHz. Insets on the right side represent the phase and intensity distributions at a cross section z=110 cm (red translucent planes).

Figure S6 shows the simulated the intensity distribution of the underwater ABV beam created by the designed quasi-3D metasurface at 30 kHz. We can observe typical

characteristics of the vortex beam in the range from 30 to 120 cm ($|p|^2>2.5$ Pa^2). The propagation distance of the underwater ABV beam (about 22λ) is larger than that (about 10λ) of the holographic algorithm. The designed underwater ABV beam with long-distance propagation is important for realizing advanced in-vivo acoustic tweezers as it can penetrate deeper to the lesion location, which can be potentially applied to broken bones removing, thrombus ablation, and nerve stimulation.
